# T46. TARGETING THE IMMUNE SYSTEM TO TREAT DEPRESSION AND NEGATIVE SYMPTOMS IN SCHIZOPHRENIA

**DOI:** 10.1093/schbul/sby016.322

**Published:** 2018-04-01

**Authors:** Carl Krynicki, Rachel Upthegrove, John Suckling, Paola Dazzan, Eileen Joyce, Stephen Lawrie, Nusrat Husain, Imran Chaudhry, Graham Dunn, Peter Jones, Danuta Lisiecka, Shon Lewis, Thomas Barnes, Stephen Williams, Stephen Hopkins, Richard Drake, Richard Smallman, Annalisa Giordano, Carmine Pariante, Bill Deakin

**Affiliations:** 1 University of Birmingham; 2 University of Cambridge; 3 Institute of Psychiatry, Psychology and Neuroscience, King’s College London; 4 UCL Institute of Neurology; 5 University of Edinburgh; 6 University of Manchester; 7 Imperial College London

## Abstract

**Background:**

Negative symptoms consist of impaired quality of life, social isolation, reduced emotional responsiveness, self-neglect and anhedonia, which have been categorised into avolition-apathy and expressive deficits sub-domains. The treatment of negative symptoms remains a challenge. Depression is commonly seen in schizophrenia and previous findings have suggested a relationship between depression and negative symptoms via the avolition-apathy sub-domain, (Barnes et al., 2016). It is possible this is the result of a common aetiology, distinct from expressive deficit or other symptoms of schizophrenia. Immune dysfunction has been implicated in both psychotic and depressive illnesses; increased circulating pro-inflammatory markers (such as IL-6, TNF-α & CRP). This suggests a novel target for treatments. A putative neuroprotective role of minocycline has been suggested via reducing microglial activation, and decrease in the production of cytokines including IL-6. Minocycline has been shown to be effective in the treatment of negative symptoms (Xiang et al., 2017) and depression (Soczynska et al., 2012). Within schizophrenia, we predict that that minocycline will lead to a longitudinal improvement in depression and the avolition-apathy sub-domain of negative symptoms

**Methods:**

Data from the BeneMin study will be presented. BeneMin recruited 207 patients with a current research diagnosis of schizophrenia within 5 years of onset and randomised to minocycline (300mg/day) or matching placebo for 12 months adjunctive to antipsychotic medication. For this analysis the primary outcome variable is the negative symptom subscale from the Positive and Negative Syndrome Scales (and broken down into avolition-apathy and expressive deficits sub-domains), Calgary Depression Scale in Schizophrenia (CDSS) and circulating IL-6, TNF-α and CRP over 4-time points 2, 6, 9, and 12 months.

**Results:**

At baseline, 40% were depressed (mean CDSS score = 5). The mean avolition-apathy PANSS score was 9.5 and expressive deficits was 9, and was comparable across placebo and minocycline arms. Preliminary results show that markers of inflammation were low in both treatment arms, compared with previous research (baseline CRP Md = 1.45, IL-6 Md = .57, TNF-α Md = 2.43) and this was comparable across depressed and non-depressed patients. TNF-α was significantly associated with expressive-deficits (B = .75, p = .005). Conversely, no marker of inflammation was associated with avolition-apathy or depression. However, in four linear mixed effect models across the 2, 6, 9 and 12-month follow-up assessments compared with placebo, minocycline had no effect on total negative symptoms, avolition-apathy, expressive deficits or depression. Further analysis stratifying patients by depression scores and markers of inflammation will be presented.

**Discussion:**

Preliminary results indicate that minocycline does not lead to a reduction in avolition-apathy or depression in early schizophrenia. This may be the result of a medicated, sample recruited within 5 years of illness onset, and low levels of depression. Future studies should target depression in psychosis as a primary aim with samples of individuals with increased inflammatory response to fully investigate minocycline’s potential in targeted intervention.

